# Supplementary material for: Polar bear energetic and behavioral strategies on land with implications for surviving the ice-free period
Source: Nat Commun. 2024 Feb 13;15:947. doi: 10.1038/s41467-023-44682-1 (PMC10864307; doi:10.1038/s41467-023-44682-1)
Supplement: Supplementary file 1 — Supplementary Information [file 41467_2023_44682_MOESM1_ESM.pdf]

Polar bear energetic and behavioral strategies on land with implications for surviving the ice-free period

Anthony M. Pagano[1*], Karyn D. Rode[1], Nicholas J. Lunn[2], David McGeachy[2], Stephen N. Atkinson[3], Sean D. Farley[4], Joy A. Erlenbach[5]†, and Charles T. Robbins[5,6].

[1] U. S. Geological Survey, Alaska Science Center, Anchorage, AK 99508, USA

[2] Wildlife Research Division, Science and Technology Branch, Environment and Climate Change Canada, Edmonton, AB T6G 2E9, Canada

[3] 226104 Melrose Road, Cooks Creek, MB, R5M 0B9, Canada

[4] Alaska Department of Fish and Game, Anchorage, AK 99518, USA

[5] School of Biological Sciences, Washington State University, Pullman, WA 99164, USA

[6] School of the Environment, Washington State University, Pullman, WA 99164, USA

† Present Address: U.S. Fish and Wildlife Service, Kodiak National Wildlife Refuge, Kodiak, AK 99615, USA

* Correspondence: apagano@usgs.gov

Supplementary Table 1. A priori models tested to explain the variation in the daily energy expenditure of 20 polar bears on land near Churchill, Manitoba, Canada. Provided are the number of parameters (k), Akaike's information criterion corrected for small sample size (AIC_c), and the difference in AIC_c between each model and the best-supported model (ΔAIC_c). Parameters tested include the sex and age class of each bear (SexAge), age as a continuous variable (Age), sex as a binary variable (Sex), mean activity and the mean proportion of the total time swimming both derived from the tri-axial accelerometer data (Activity, Swim; respectively), mean movement rate derived from the GPS location data (MovementRate), the percent body mass change between the initial capture and recapture (MassChange), the amount of body fat at the initial capture based on deuterium dilution (Fat), and whether the bear was pregnant based on its blood serum progesterone level (Pregnant).

Model	k	AIC_c	ΔAIC_c
SexAge + Activity	6	198.3	0.0
SexAge + MovementRate	6	198.5	0.2
SexAge × Activity + MassChange	10	200.4	2.1
SexAge + MassChange + Activity	7	201.8	3.5
Activity	3	201.9	3.6
SexAge × Activity	9	202.2	3.8
SexAge + Activity + Pregnant	7	202.6	4.2
SexAge + Fat + Activity	7	202.9	4.6
Age + Sex + Activity + Pregnant	6	203.3	5
SexAge + Swim	6	203.6	5.3
Activity + Fat	4	203.8	5.5
Age + Activity	4	204.6	6.3
Activity + MassChange	4	204.7	6.4
Age + Sex + Activity	5	207.2	8.9
SexAge + Fat + MassChange + Activity	8	207.4	9.1
SexAge + Fat + MassChange + Activity + Pregnant	9	213.0	14.7
Null	2	214.4	16.1
SexAge	5	214.5	16.2

Supplementary Table 2. A priori models tested to explain the variation in the percent change in body mass of 19 polar bears that lost mass while on land near Churchill, Manitoba, Canada. Provided are the number of parameters (k), Akaike's information criterion corrected for small sample size (AIC_c), and the difference in AIC_c between each model and the best-supported model (ΔAIC_c). Parameters tested include the sex and age class of each bear (SexAge), accelerometer-derived measures of mean activity, mean proportion of the total time eating, and the mean proportion of the total time swimming (Activity, Eat, and Swim; respectively), mean movement rate derived from the GPS location data (MovementRate), the mass specific daily energy expenditure (DEE), the amount of body fat at the initial capture based on deuterium dilution (Fat), and whether the bear was pregnant based on its blood serum progesterone level (Pregnant).

Model	k	AIC_c	ΔAIC_c
Null	2	-87.4	0.0
DEE	3	-86.0	1.4
Activity	3	-84.7	2.7
Swim	3	-84.6	2.8
MovementRate	3	-84.6	2.8
Eat	3	-84.5	2.9
Fat + MovementRate	4	-81.3	6.1
SexAge × MovementRate	9	-80.9	6.5
SexAge	5	-80.4	7.0
SexAge + MovementRate	6	-76.1	11.3
SexAge + Fat	6	-71.8	15.6
SexAge + Fat + Activity	7	-70.9	16.5
SexAge × Activity	9	-67.4	20.0
SexAge + Fat + MovementRate + Pregnant	8	-66.7	20.7

Supplementary Table 3. Body mass and composition of 20 polar bears on land near Churchill, Manitoba, Canada based on deuterium dilution from their initial capture and their recapture 19 – 23 days later. Estimates of lean body mass (LBM) and fat mass were derived using the equations of Farley and Robbins[56] ('Farley and Robbins') and Cattet et al.[64] ('Cattet').

Bear	Initial Mass (kg)	Initial LBM (kg)		Initial fat mass (kg)		Final Mass (kg)	Final LBM (kg)		Final fat mass (kg)	
		Farley and Robbins	Cattet	Farley and Robbins	Cattet		Farley and Robbins	Cattet	Farley and Robbins	Cattet
X17517	410	294	305	116	105	387	282	291	105	95
X19911	273	176	186	97	87	242	147	157	95	85
X33823	235	170	176	65	59	267	169	179	98	88
X33824	215	150	156	64	58	204	140	146	64	58
X33935	182	109	117	73	65	165	96	103	68	61
X33939	202	130	138	71	64	192	121	128	71	64
X33653	165	112	117	53	48	157	106	111	50	45
X33934	262	130	145	131	116	242	124	137	118	105
X33928	263	145	158	118	105	244	130	143	114	101
X33936	209	133	141	76	68	191	121	129	69	62
X33938	259	142	155	117	104	245	129	142	116	103
X33991	155	97	103	57	51	139	98	102	41	37
X33954	166	101	108	65	58	150	102	107	47	43
X33851	273	176	186	97	87	243	169	176	74	67
X33410	318	167	184	151	134	305	175	189	129	115
X33712	296	153	169	143	126	273	156	169	117	104
X32415	446	308	321	138	125	414	289	300	124	112
X19842	526	322	344	203	182	490	308	327	182	163
X33302	484	315	332	169	152	451	310	323	141	127
X32422	583	350	375	233	208	550	361	380	187	169

Supplementary Table 4. Dose rates of oxygen 18 (^{18}O) and deuterated water (^{2}H) in doubly-labeled water doses of 20 polar bears on land near Churchill, Manitoba, Canada. Oxygen-18 was enriched at 98.4% (Isoflex USA, San Francisco, CA). Deuterated water was enriched at 99.8% (Sigma Aldrich, Inc., St. Louis, MO). The doubly-labeled water was made isotonic with 0.9% NaCl.

Year	Bear	Age	Sex	^{18}O dose ($g•kg^{-1}$)	^{2}H dose ($g•kg^{-1}$)
2019	X17517	Adult	Male	0.41	0.21
2019	X19911	Adult	Female	0.47	0.24
2021	X33823	Subadult	Male	0.41	0.21
2021	X33824	Subadult	Male	0.30	0.15
2021	X33935	Subadult	Female	0.26	0.13
2021	X33939	Subadult	Female	0.36	0.18
2021	X33653	Adult	Female	0.64	0.32
2021	X33934	Adult	Female	0.37	0.18
2021	X33928	Adult	Female	0.37	0.19
2021	X33936	Adult	Female	0.47	0.23
2021	X33938	Adult	Female	0.37	0.19
2022	X33991	Subadult	Female	0.35	0.18
2022	X33954	Subadult	Female	0.35	0.17
2022	X33851	Subadult	Male	0.36	0.18
2022	X33410	Adult	Female	0.35	0.18
2022	X33712	Adult	Female	0.36	0.18
2022	X32415	Adult	Male	0.32	0.16
2022	X19842	Adult	Male	0.29	0.14
2022	X33302	Adult	Male	0.34	0.17
2022	X32422	Adult	Male	0.31	0.16

Supplementary Table 5. Video recording schedules used by GPS-equipped video camera collars deployed on polar bears on land near Churchill, Manitoba, Canada.

Year	Time of Day[a]	Video recording duty cycle
2019	Continuous	10 sec : 5 min
2021	5:00 am – 6:00 pm	5 sec : 2 min
2022[b]	7:11 pm – 8:11 am	5 sec : 2 min
2022[c]	6:11 am – 7:11 pm	5 sec : 2 min

[a] Times are Central Daylight Time.
[b] Due to a programming error this was the initial recording schedule of collars until 13 September.
[c] Recording schedule of collars after 13 September.

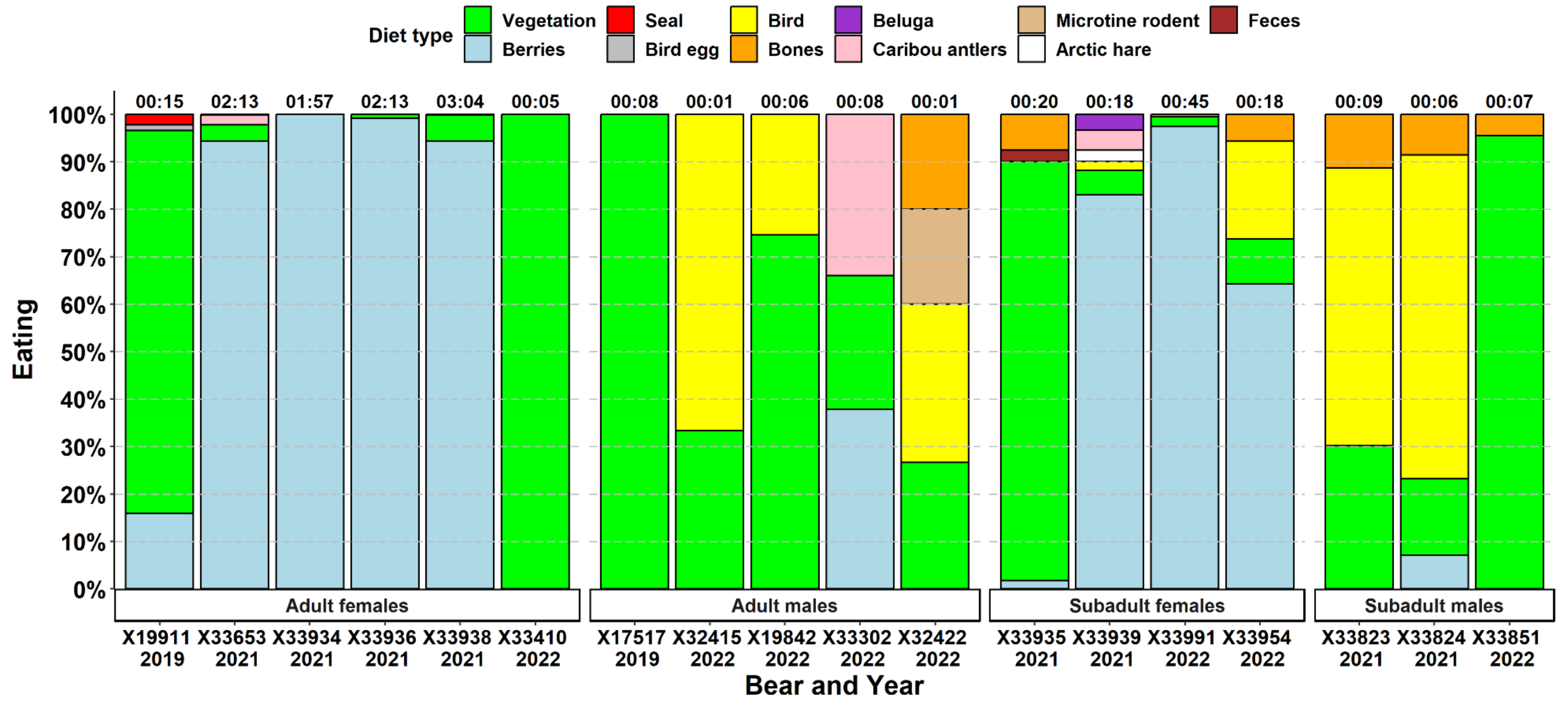

Supplementary Figure 1. Percent time engaged in eating behaviors by polar bears on land near Churchill, Manitoba, Canada. Percent time eating is shown relative to the diet type consumed based on the video footage from 18 polar bears recorded during daylight hours. The numbers above each vertical bar refers to the total number of hours and minutes (hh:mm) that bears were recorded eating within the duty cycled video footage.

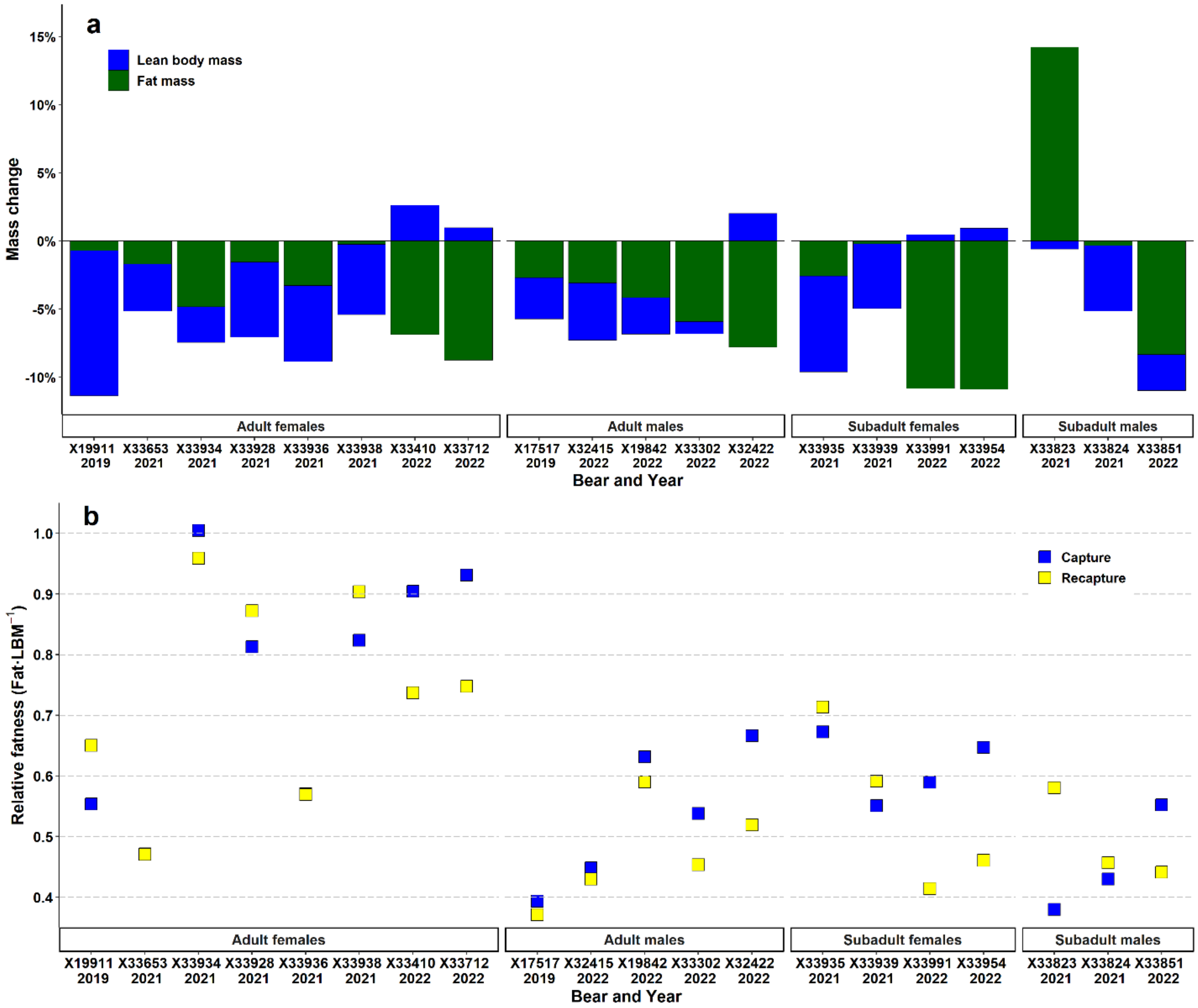

Supplementary Figure 2. Changes in the body composition of 20 polar bears on land near Churchill, Manitoba, Canada. Body composition was measured using deuterium dilution and measures of body mass at the initial capture and recapture 19 – 23 days later. (**a**) percent change in lean body mass (LBM) and fat mass of bears from their initial capture to their recapture, and (**b**) relative fatness (body fat•LBM^{-1}) of bears at their initial capture and recapture.
