# Supplementary material for: Polar bear energetic and behavioral strategies on land with implications for surviving the ice-free period
Source: Nat Commun. 2024 Feb 13;15:947. doi: 10.1038/s41467-023-44682-1 (PMC10864307; doi:10.1038/s41467-023-44682-1)
Supplement: Supplementary file 3 — Description of Additional Supplementary Files [file 41467_2023_44682_MOESM3_ESM.pdf]

Description of Additional Supplementary Files:

Supplementary Movie 1: Animation of movements from 20 polar bears on land. Adult females (green circles, n = 8), subadult females (orange circles, n = 4), subadult males (yellow circles, n = 3), and adult males (purple circles, n = 5) on land near Churchill, Manitoba, Canada. Movements were derived from GPS-enabled video camera collars over 19 – 23 days in 2019 – 2022. Hourly movements from GPS location data are shown in relation to the Wapusk National Park boundary (dashed line). Datetimes in GMT.

Supplementary Movie 2: Video from a camera collar on an adult female polar bear (X33653) while on land near Churchill, Manitoba in September 2021. This video shows X33653 on 4 September 2021 chewing on caribou antlers and eating berries. The video also shows her on 8 September 2021 eating berries and on 9 September 2021 eating grasses. Datetimes in GMT.

Supplementary Movie 3: Video from a camera collar on a subadult male polar bear (X33823) while on land near Churchill, Manitoba in September 2021. This video shows X33823 on 3 September 2021 eating a bird carcass. Datetimes in GMT.

Supplementary Movie 4: Video from a camera collar on an adult female polar bear (X19911) while swimming near Churchill, Manitoba in September 2019. This video shows X19911 on 6 September 2019 swimming while carrying a seal carcass. Datetimes in GMT.

Supplementary Movie 5: Video from a camera collar on a subadult female polar bear (X33939) while swimming near Churchill, Manitoba in August 2021. This video shows

X33939 on 29 August 2021 swimming near and eating from a beluga carcass. Datetimes in GMT.
