# Supplementary material for: Polar bear energetic and behavioral strategies on land with implications for surviving the ice-free period
Source: Nat Commun. 2024 Feb 13;15:947. doi: 10.1038/s41467-023-44682-1 (PMC10864307; doi:10.1038/s41467-023-44682-1)
Supplement: Supplementary file 9 — Reporting Summary [file 41467_2023_44682_MOESM9_ESM.pdf]

nature portfolio

Corresponding author(s): Anthony M. Pagano

Last updated by author(s): Dec 18, 2023

Reporting Summary

Nature Portfolio wishes to improve the reproducibility of the work that we publish. This form provides structure for consistency and transparency in reporting. For further information on Nature Portfolio policies, see our Editorial Policies and the Editorial Policy Checklist.

Statistics

For all statistical analyses, confirm that the following items are present in the figure legend, table legend, main text, or Methods section.

n/a	Confirmed	
☐	☒	The exact sample size (*n*) for each experimental group/condition, given as a discrete number and unit of measurement
☒	☐	A statement on whether measurements were taken from distinct samples or whether the same sample was measured repeatedly
☐	☒	The statistical test(s) used AND whether they are one- or two-sided<br>*Only common tests should be described solely by name; describe more complex techniques in the Methods section.*
☐	☒	A description of all covariates tested
☐	☒	A description of any assumptions or corrections, such as tests of normality and adjustment for multiple comparisons
☐	☒	A full description of the statistical parameters including central tendency (e.g. means) or other basic estimates (e.g. regression coefficient) AND variation (e.g. standard deviation) or associated estimates of uncertainty (e.g. confidence intervals)
☐	☒	For null hypothesis testing, the test statistic (e.g. *F*, *t*, *r*) with confidence intervals, effect sizes, degrees of freedom and *P* value noted<br>*Give P values as exact values whenever suitable.*
☒	☐	For Bayesian analysis, information on the choice of priors and Markov chain Monte Carlo settings
☒	☐	For hierarchical and complex designs, identification of the appropriate level for tests and full reporting of outcomes
☒	☐	Estimates of effect sizes (e.g. Cohen's *d*, Pearson's *r*), indicating how they were calculated

Our web collection on statistics for biologists contains articles on many of the points above.

Software and code

Policy information about availability of computer code

Data collection	Expedata acquisition and analysis software (Sable Systems International).
Data analysis	SAS version 9.4 (SAS Institute Inc.), R (v. 4.3.2): A language and environment for statistical computing (R Core Team), R packages: ‘ggplot2’, ‘ggmap’, ‘maptools’, ‘randomForest’, ‘plyr’, ‘dplyr’,‘move’.

For manuscripts utilizing custom algorithms or software that are central to the research but not yet described in published literature, software must be made available to editors and reviewers. We strongly encourage code deposition in a community repository (e.g. GitHub). See the Nature Portfolio guidelines for submitting code & software for further information.

Data

Policy information about availability of data

All manuscripts must include a data availability statement. This statement should provide the following information, where applicable:

- Accession codes, unique identifiers, or web links for publicly available datasets
- A description of any restrictions on data availability
- For clinical datasets or third party data, please ensure that the statement adheres to our policy

The data associated with this paper have been publicly released at: https://doi.org/10.5066/P9A7ITFH

Research involving human participants, their data, or biological material

Policy information about studies with human participants or human data. See also policy information about sex, gender (identity/presentation), and sexual orientation and race, ethnicity and racism.

Reporting on sex and gender	N/A
Reporting on race, ethnicity, or other socially relevant groupings	N/A
Population characteristics	N/A
Recruitment	N/A
Ethics oversight	N/A

Note that full information on the approval of the study protocol must also be provided in the manuscript.

Field-specific reporting

Please select the one below that is the best fit for your research. If you are not sure, read the appropriate sections before making your selection.

☐ Life sciences ☐ Behavioural & social sciences ☒ Ecological, evolutionary & environmental sciences

For a reference copy of the document with all sections, see nature.com/documents/nr-reporting-summary-flat.pdf

Ecological, evolutionary & environmental sciences study design

All studies must disclose on these points even when the disclosure is negative.

Study description

We measured the energy expenditure, diet, behavior, activity, movement rate, blood chemistry, and body composition of 20 different polar bears (Ursus maritimus) on land near Churchill, Manitoba, Canada over 19 – 23 days. Daily energy expenditure and body composition were measured using doubly-labeled water and isotopic dilution. GPS–equipped video camera collars with tri-axial accelerometers were used to determine diet, activity, behavior, and movement rates, which in turn were used to assess the causes of variation in daily energy expenditure. We evaluated factors influencing changes in body mass using video-derived observations of foraging, measures of blood biochemistry, and estimates of daily energy expenditure. Individual variation across all bears and within sex and age classes was estimated based on the coefficient of variation (CV). We used multiple linear regression with the mass-specific daily energy expenditure (DEE) as the response variable and the percent time active or swimming derived from the tri-axial accelerometer data, movement rate derived from the GPS location data, percent initial body fat, percent body mass change, whether bears were pregnant based on their serum progesterone, and sex and age class as predictors. We similarly used multiple linear regression with the percent body mass change as the response variable and activity, swimming, and eating derived from the tri-axial accelerometer data, mass-specific DEE, movement rate derived from the GPS location data, percent initial body fat, whether bears were pregnant, and sex and age class as the predictors. We generated a priori sets of candidate models and used an information-theoretic approach based on Akaike's information criterion corrected for small sample size (AICc). We considered top-ranked models as those with ΔAICc values < 2.0.
All analyses were conducted in R with $\alpha = 0.05$. Means are presented ± 1 SE.

Research sample

We collected data from 20 polar bears (Ursus maritimus) over 19 – 23 days. This consisted of 8 solitary adult females, 5 adult males, 4 subadult females, and 3 subadult males. Bear ages ranged from 2 - 21 years old. This sample size was determined to allow us to evaluate how energy expenditure, diet, behavior, and activity varied by sex and age class, which will provide important baselines for predicting the ability of polar bears to survive on land for increasing durations due to future climate change.

Sampling strategy

We collected data from 8 solitary adult female, 5 adult male, 4 subadult female, and 3 subadult male polar bears. This sample size was chosen to enable comparisons among sex and age classes. Due to the costs of the doubly-labeled water, GPS-enabled video camera collars, and the helicopter that was needed to immobilize bears, this was the largest sample size that was feasible given our goals of evaluating energy expenditure, diet, behavior, activity, and changes in body mass and composition among multiple polar bear age and sex classes while on land.

Data collection

Data was primarily collected by A.M.P. with assistance from K.D.R., N.J.L., D.M., S.N.A., and J.A.E. We inserted either an external jugular or cephalic catheter to facilitate blood sampling and administration of isotopes. We took a blood sample at the time of the initial capture to serve as a baseline measure of oxygen-18 (18O) and deuterated water (2H). The bear was then injected intravenously with a precisely weighed dose containing 0.26 – 0.64 g/kg of 98.4% enriched 18O (Isoflex USA, San Francisco, CA) and 0.13 – 0.32 g/kg of 99.8% enriched 2H (Sigma Aldrich, Inc., St. Louis, MO) with NaCl added to make it 0.9% isotonic and sterilized using a 0.2 µ Millipore filter (Corning, Inc., Corning, NY). On injection, the syringe was backwashed with blood three times to ensure all the DLW had been injected into the bear. The bear was kept immobilized for 2 hr after the injection of DLW to allow isotope equilibration. We collected serial blood samples 30, 60, 90, and 120 min after dosing to evaluate equilibration curves. The bears were weighed using an electronic load cell suspended from an aluminum tripod. We recaptured bears 19 – 23 days after initial capture to obtain a blood sample to measure final enrichment. Similar to the initial capture, following immobilization, we inserted either an external jugular or cephalic catheter to facilitate blood sampling and administration of isotopes. An initial blood sample was collected

to measure final enrichment after which bears were dosed with 0.10 – 0.11 g/kg of 99.8% enriched 2H (Sigma Aldrich, Inc.) made isotonic with 0.9% NaCl and sterilized using a 0.2 µ Millipore filter. On injection, the syringe was backwashed with blood three times to ensure all the 2H had been injected into the bear and serial blood samples were collected 30, 60, 90, and 120 min after dosing. We measured the respiratory exchange ratio (RER) of bears from respired samples collected by placing a mask (Smiths Medical Inc., Dublin, OH) over the snout of each bear. The mask was attached to a two-way valve with ports for inhalation and exhalation (Hans Rudolph Inc., Shawnee, KS), which allowed the bear to inhale fresh air while exhaling into a 25-liter Douglas bag (Harvard Apparatus, Holliston, MA). We deployed Global Positioning System (GPS)-enabled video camera collars (Vertex Plus collar with camera option, Vectronic Aerospace GmbH, Berlin, Germany) on the same individuals that were dosed with DLW. Duty cycles and schedules of video cameras varied among years with cameras turning on for 10 sec every 5 min in 2019 and 5 sec every 2 min in 2021 and 2022. Collars recorded a GPS fix every 5 min, which were stored in the collar's nonvolatile memory and downloaded upon recovery. Collars also transmitted a subset of these fixes via the Iridium satellite system. Additionally, collars measured tri-axial acceleration at 16 Hz (range ±4 g).

Timing and spatial scale	Data was collected from 2 individuals in 2019, 9 individuals in 2021, and 9 individuals in 2022. Each individual was studied for 19 – 23 days. In each year, data was collected from late August to mid-September. This timing was chosen as it represents a relative midpoint between the timing of arrival of polar bears on land from Western Hudson Bay and the timing of their departure back on to the sea ice. Additionally, earlier in the summer on land in northern Manitoba temperatures can be too warm to safely immobilize polar bears from a helicopter.
Data exclusions	Video data was excluded from 2 collars that failed within 1- and 4-days post-capture. No other data was excluded from analysis.
Reproducibility	Given the unique nature of this study, reproducibility is not feasible.
Randomization	We sought to randomly sample wild polar bears within Wapusk National Park, Manitoba, Canada, while also attempting to have a relatively balanced sample of adult females, adult males, and subadults. We also sought to geographically distribute our sample throughout Wapusk National Park by sampling bears both along the coast, inland, and along a north-south gradient within the flight range of our helicopter.
Blinding	Blinding was not possible in this study. Nevertheless we sought to randomly sample wild polar bears within Wapusk National Park, Manitoba, Canada, while also attempting to have a relatively balanced sample of adult females, adult males, and subadults that were geographically distributed throughout Wapusk National Park.

Did the study involve field work? ☒ Yes ☐ No

Field work, collection and transport

Field conditions	Data were collected from polar bears captured on land in Wapusk National Park, Manitoba, Canada. Wapusk National Park covers 11,475 square km. It consists of a mixture of arctic tundra, muskeg, peatlands, and boreal forest and borders Hudson Bay. Rainfall in August and September averages of 67 mm per month. Average temperatures in August and September range from 3.2 - 16.7°C
Location	Data were collected from polar bears captured on land in Wapusk National Park, Manitoba, Canada. This area has an elevation < 200 m. The bounding coordinates of the study area included: West Bounding Coordinate: -95.019, East Bounding Coordinate: -92.419, North Bounding Coordinate: 60.199, and South Bounding Coordinate: 57.402
Access & import/export	Access to habitats and animal handling procedures were approved by Parks Canada Wapusk National Park Research and Collection Permits #WAP-2020-37418 (Issued: 29 October 2020), WAP-2020-36578 (Issued: 22 August 2020), and WAP2019-32265 (Issued: 15 May 2019), Manitoba Species at Risk/Wildlife Scientific Permits #SAR21014 (Issued: 30 June 2021) and SAR20021 (Issued 12 August 2020), and an Exemption Permit issued under the authority of section 21 of the Wildlife Act by the Department of Environment, Nunavut (Issued: 13 September 2021). Blood samples were exported under CITES Permit 22CA03597/CWHQ (Issued: 21 October 2022) and Marine Mammal Research Permits: MA82088B-1 (Issued: 21 July 2020) and MA690038-17 (Issued: 21 February 2013).
Disturbance	We made efforts to avoid disturbance to other wildlife and people by avoiding human settlements and by flying at high altitudes when not actively searching for bears.

Reporting for specific materials, systems and methods

We require information from authors about some types of materials, experimental systems and methods used in many studies. Here, indicate whether each material, system or method listed is relevant to your study. If you are not sure if a list item applies to your research, read the appropriate section before selecting a response.

Materials & experimental systems

n/a	Involved in the study
☒	☐ Antibodies
☒	☐ Eukaryotic cell lines
☒	☐ Palaeontology and archaeology
☐	☒ Animals and other organisms
☒	☐ Clinical data
☒	☐ Dual use research of concern
☒	☐ Plants

Methods

n/a	Involved in the study
☒	☐ ChIP-seq
☒	☐ Flow cytometry
☒	☐ MRI-based neuroimaging

Animals and other research organisms

Policy information about studies involving animals; ARRIVE guidelines recommended for reporting animal research, and Sex and Gender in Research

Laboratory animals

The study did not involve laboratory animals.

Wild animals

Polar bears (Ursus maritimus) were located from a helicopter and captured using standard chemical immobilization techniques. We collected data from 8 adult female, 5 adult male, 4 subadult female, and 3 subadult male polar bears. Specific ages of each individual are provided in Table 1. We classified adults as ≥5 years old and subadults as independent bears that were 2–4 years old. All individuals were recaptured 19 – 23 days after their initial capture to measure their energy expenditure, changes in body mass and body composition, blood biochemistry, and to remove and recover their GPS–equipped video camera collar containing a tri-axial accelerometer and temperature sensor. After the collection of physiological samples, all animals were allowed to recover from immobilization and released back into the wild.

Reporting on sex

To the extent possible, we attempted to collect data from both sexes to evaluate potential differences in energy expenditure, diet, behavior, activity, and changes in body mass and composition based on sex. Ultimately, we collected data from 12 female (8 adult female) and 8 male (5 adult male) polar bears. In our analyses, we tested for the effects of age class and sex on energy expenditure and changes in body mass.

Field-collected samples

Blood was collected in 10 ml glass evacuated tubes without anticoagulants (Serum Vacutainer, Becton Dickinson, Franklin Lakes, NJ or Monoject noncoated tubes, Cardinal Health, Dublin, OH) and centrifuged to separate serum from red blood cells. Serum was stored frozen in 2 ml cryogenic vials (Corning, Inc.) at -80°C until analysis. Water was extracted from each serum sample by vacuum sublimation. Specific activity of 18O and 2H were determined by wavelength-scanned cavity ring-down spectroscopy (Metabolic Solutions, Inc., Nashua, NH). Serum progesterone (P4) levels of adult female polar bears were measured by radioimmunoassay (ImmuChem™ Progesterone 125I, MP Biomedicals, LLC, Santa Ana, CA) by either the Endocrine Service Laboratory at the University of Saskatchewan (Saskatoon, SK) or the Endocrinology Laboratory at the Animal Health Diagnostic Center (Cornell University, Ithaca, NY). Serum samples of all bears were analyzed for blood urea nitrogen (BUN) and creatinine using an Abaxis Vetscan VS2 chemistry analyzer (Abaxis, Inc., Union City, CA). We measured the respiratory exchange ratio (RER) of bears from respired samples collected by placing a mask (Smiths Medical Inc., Dublin, OH) over the snout of each bear. The mask was attached to a two-way valve with ports for inhalation and exhalation (Hans Rudolph Inc., Shawnee, KS), which allowed the bear to inhale fresh air while exhaling into a 25-liter Douglas bag (Harvard Apparatus, Holliston, MA). We calculated the RER from these breath samples using an O2 and CO2 analyzer (FOXBOX; Sable Systems International, Las Vegas, NV).

Ethics oversight

All procedures were approved by the Animal Care and Use Committees of the U.S. Geological Survey, Alaska Science Center and the Environment and Climate Change Canada Prairie and Northern Region. Research was approved under permits from Parks Canada Wapusk National Park Research and Collection Permits (#WAP-2020-37418, WAP-2020-36578), Manitoba Species at Risk/Wildlife Scientific Permits (#SAR21014, SAR20021), and an Exemption Permit issued by the Department of Environment, Nunavut.

Note that full information on the approval of the study protocol must also be provided in the manuscript.

Plants

Seed stocks

N/A

Novel plant genotypes

N/A

Authentication

N/A
